# Vero cell-adapted SARS-CoV-2 strain shows increased viral growth through furin-mediated efficient spike cleavage

**DOI:** 10.1128/spectrum.02859-23

**Published:** 2024-02-28

**Authors:** Shohei Minami, Tomohiro Kotaki, Yusuke Sakai, Shinya Okamura, Shiho Torii, Chikako Ono, Daisuke Motooka, Rina Hamajima, Ryotaro Nouda, Jeffery A. Nurdin, Moeko Yamasaki, Yuta Kanai, Hirotaka Ebina, Yusuke Maeda, Toru Okamoto, Taro Tachibana, Yoshiharu Matsuura, Takeshi Kobayashi

**Affiliations:** 1 Department of Virology, Research Institute for Microbial Diseases, Osaka University, Osaka, Japan; 2 Department of Pathology, National Institute of Infectious Diseases, Tokyo, Japan; 3 Virus Vaccine Group, BIKEN Innovative Vaccine Research Alliance Laboratories, Institute for Open and Transdisciplinary Research Initiatives, Osaka University, Osaka, Japan; 4 The Research Foundation for Microbial Diseases of Osaka University, Suita, Osaka, Japan; 5 Laboratory of Virus Control, Research Institute for Microbial Diseases, Osaka University, Osaka, Japan; 6 Center for Infectious Disease Education and Research, Osaka University, Osaka, Japan; 7 Department of Infection Metagenomics, Research Institute for Microbial Diseases, Osaka University, Osaka, Japan; 8 Center for Advanced Modalities and DDS, Osaka University, Osaka, Japan; 9 Laboratory of Viral Dynamism Research, Research Institute for Microbial Diseases Osaka University, Osaka, Japan; 10 Institute for Advanced Co-creation Studies, Research Institute for Microbial Diseases Osaka University, Osaka, Japan; 11 Cell Engineering Corporation, Osaka, Japan; 12 Department of Bioengineering, Graduate School of Engineering, Osaka Metropolitan University, Osaka, Japan; Karolinska Institutet, Stockholm, Sweden

**Keywords:** severe acute respiratory syndrome virus-2 (SARS-CoV-2), spike (S) protein, viral replication, infection, pathogenicity

## Abstract

Severe acute respiratory syndrome coronavirus 2 (SARS-CoV-2) utilizes several host proteases to cleave the spike (S) protein to enter host cells. SARS-CoV-2 S protein is cleaved into S1 and S2 subunits by furin, which is closely involved in the pathogenicity of SARS-CoV-2. However, the effects of the modulated protease cleavage activity due to S protein mutations on viral replication and pathogenesis remain unclear. Herein, we serially passaged two SARS-CoV-2 strains in Vero cells and characterized the cell-adapted SARS-CoV-2 strains *in vitro* and *in vivo*. The adapted strains showed high viral growth, effective S1/S2 cleavage of the S protein, and low pathogenicity compared with the wild-type strain. Furthermore, the viral growth and S1/S2 cleavage were enhanced by the combination of the Δ68–76 and H655Y mutations using recombinant SARS-CoV-2 strains generated by the circular polymerase extension reaction. The recombinant SARS-CoV-2 strain, which contained the mutation of the adapted strain, showed increased susceptibility to the furin inhibitor, suggesting that the adapted SARS-CoV-2 strain utilized furin more effectively than the wild-type strain. Pathogenicity was attenuated by infection with effectively cleaved recombinant SARS-CoV-2 strains, suggesting that the excessive cleavage of the S proteins decreases virulence. Finally, the high-growth-adapted SARS-CoV-2 strain could be used as the seed for a low-cost inactivated vaccine; immunization with this vaccine can effectively protect the host from SARS-CoV-2 variants. Our findings provide novel insights into the growth and pathogenicity of SARS-CoV-2 in the evolution of cell-cell transmission.

**IMPORTANCE:**

The efficacy of the S protein cleavage generally differs among the SARS-CoV-2 variants, resulting in distinct viral characteristics. The relationship between a mutation and the entry of SARS-CoV-2 into host cells remains unclear. In this study, we analyzed the sequence of high-growth Vero cell-adapted SARS-CoV-2 and factors determining the enhancement of the growth of the adapted virus and confirmed the characteristics of the adapted strain by analyzing the recombinant SARS-CoV-2 strain. We successfully identified mutations Δ68-76 and H655Y, which enhance viral growth and the S protein cleavage by furin. Using recombinant viruses enabled us to conduct a virus challenge experiment *in vivo*. The pathogenicity of SARS-CoV-2 introduced with the mutations Δ68-76, H655Y, P812L, and Q853L was attenuated in hamsters, indicating the possibility of the attenuation of excessive cleaved SARS-CoV-2. These findings provide novel insights into the infectivity and pathogenesis of SARS-CoV-2 strains, thereby significantly contributing to the field of virology.

## INTRODUCTION

Severe acute respiratory syndrome coronavirus 2 (SARS-CoV-2), first detected in China in 2019 (1), causes the coronavirus disease 2019 (COVID-19) with severe acute respiratory syndrome and other symptoms, which has become a global pandemic. The virus frequently mutates and changes its virological characteristics. Further research on mechanisms underlying viral replication and vaccine development is therefore imperative to overcome this pandemic. The spike (S) protein, located on the outer region of the viral envelope, plays a crucial role in mediating virus entry into the host cells (2). The S protein is a highly antigenic trimetric protein that has undergone frequent mutations during recent epidemics. The S protein attaches to the angiotensin-converting enzyme 2 (ACE2), the receptor for SARS-CoV-2, which is then cleaved by host proteases, essential for virus entry into host cells (3–7). In this process, the S protein is divided into two domains, namely S1 and S2. The S1 domain contains an N-terminal domain (NTD) and receptor binding domain (RBD), while the S2 contains a fusion peptide (FP) and several other functional protein domains (8) and is further cleaved into S2′ by host proteases, exposing the FP to the surface of viral particles, facilitating membrane fusion. Altogether, the RBD binds to the receptor, ACE2, and the S protein is processed by several host proteases, mediating membrane fusion. The viral genome is released into the cytoplasm upon membrane fusion, followed by viral replication.

The cleavage of the S protein is mediated primarily by three host proteases (8). First, transmembrane protease serine 2 (TMPRSS2) localizes to the cell membrane, facilitating virus entry into host cells (9, 10). Second, cathepsin-L (CTSL), which localizes to endosomes, cleaves the S protein under low pH conditions and mediates virus entry during endocytosis (6, 11). TMPRSS2 and CTSL approach the cleavage sites at both S1/S2 and S2′ junctions and cleave the S protein (5). The fusion of the viral envelope occurs with the cellular membrane, either at the cell surface or with an endosomal membrane depending on the host proteases present. For instance, in the presence of TMPRSS2, fusion occurs at the cell surface, while in the absence of proteases at the cell surface, the virus is internalized and uses the intracellular CTSL to activate its fusion. Third, furin localizes primarily to the endoplasmic reticulum-Golgi network and cleaves at the S1/S2 cleavage site (12, 13). SARS-CoV-2 possesses a multi-basic S1/S2 cleavage site processed by furin, drastically increasing viral infectivity (4, 14, 15) but is not essential for infection and cell-cell fusion (16). Even if the site is frequently mutated, the cleavage site by furin has been conserved as functional (17), illustrating the importance of the furin cleavage site. Recombinant SARS-CoV-2 viruses with no furin cleavage sites showed attenuated virulence and decreased transmission *in vivo*, indicating the importance of this cleavage in viral infections (18, 19). Mutations in the RBD of the S protein affect viral infectivity by modulating the affinity between the S protein and ACE2 (20). Similarly, mutations in the NTD affect infectivity by modulating S protein cleavage. A recent report indicated that the deletion 69–70 (Δ69–70) of the NTD in the Alpha strain (B.1.1.7 lineage) increased furin-dependent S1/S2 cleavage of the S protein (21). However, the detailed role of the Δ69–70 mutation remains unclear as an analysis using recombinant SARS-CoV-2 has not yet been conducted. Considering that the Δ69–70 mutation is also conserved in several sublineages of the Omicron strain (B.1.1.529 lineage), currently the most relevant variant, the function of this deletion remains to be fully analyzed (22–24). In addition, a few antibodies that target NTD have shown enhanced SARS-CoV-2 infection by altering the conformation of the S protein, keeping RBD “up” (25). Thus, NTDs may modulate receptor binding and conformational changes in proteins.

Several studies have been conducted on the S protein through serial passaging of SARS-CoV-2 in cell lines, which is a common approach for dissecting the viral replication mechanism since the passaged virus acquires mutations presumably involved in viral replication. As SARS-CoV-2 grows well in Vero cells, studies on SARS-CoV-2 adaptation in Vero cells have been reported; indeed, SARS-CoV-2 adaptation to VeroE6 cells induced an amino acid deletion around the multibasic furin cleavage site (R685), making the S protein uncleavable by furin (26–29). A similar study reported the adaptation of a recombinant vesicular stomatitis virus (VSV) with the S protein of SARS-CoV-2 in Vero cells (30). The adapted recombinant VSV acquires an H655Y mutation in the S protein. H655Y increases cleavage of the S protein, increases cell-cell fusion activity by furin (31), enhances viral transmission *in vivo* (32, 33), and increases dependence on CTSL usage (34). Although analyses of the H655Y mutation have been conducted, recombinant SARS-CoV-2 has not been used in these studies; thus, the functions of point-mutated S proteins in authentic viruses remain unclear. Moreover, as studies using recombinant SARS-CoV-2 and animal models are limited, the effect of point mutations on pathogenicity is not fully understood.

In this study, we established Vero cell-adapted SARS-CoV-2 strains showing increased viral growth *in vitro*, enhanced S1/S2 cleavage, and reduced virulence *in vivo*. The adapted strains have two intriguing mutations, namely Δ68–76 and H655Y. Here, we clarified the function of these mutations in terms of virological characteristics and pathogenicity, using recombinant SARS-CoV-2 and a hamster model.

## RESULTS

### Virological characterization of Vero cell-adapted SARS-CoV-2 strains

**Fig 1:**
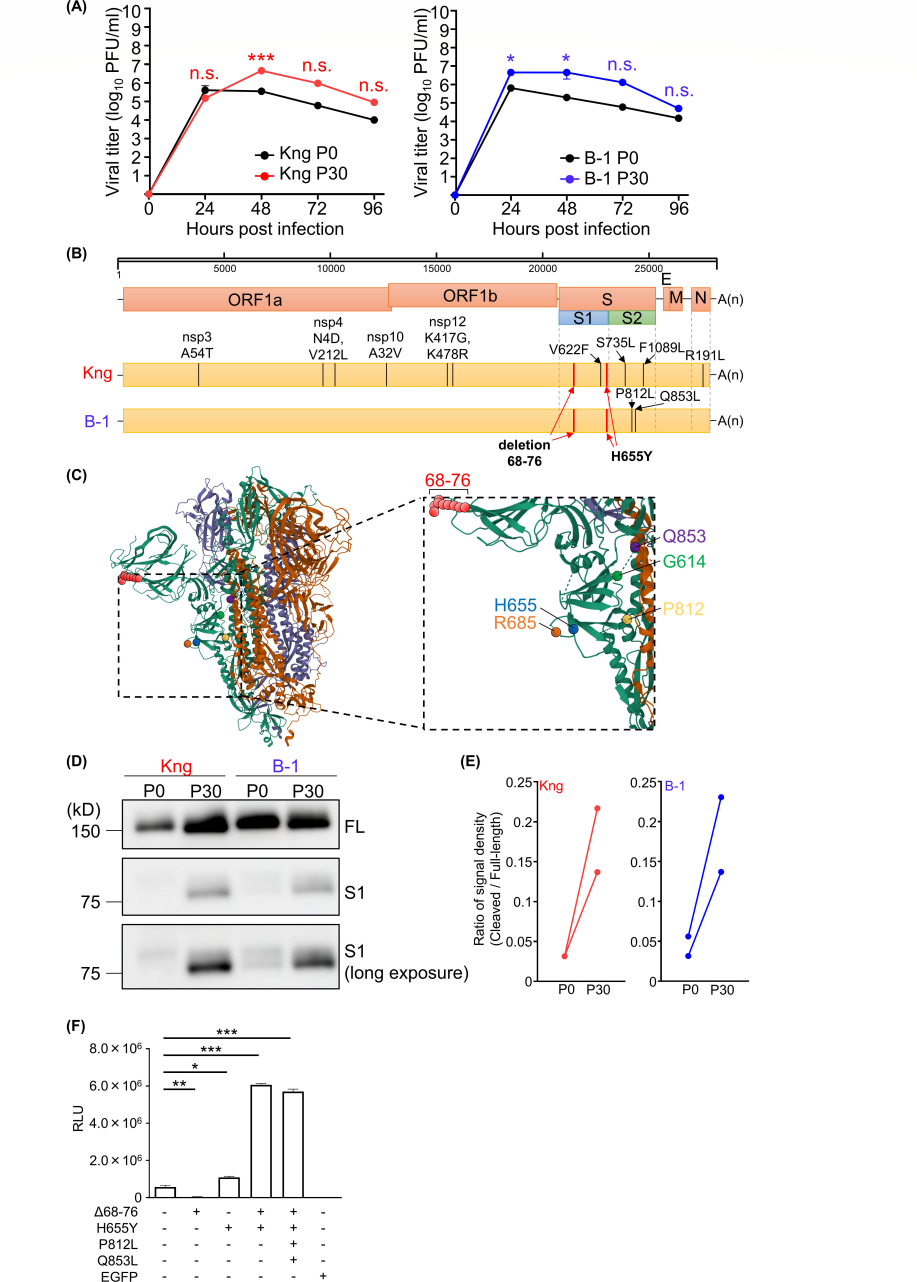
Adaptation of SARS-CoV-2 for growth in Vero cells through serial passages. (**A**) The growth kinetics of Kng or B-1 strain-based adapted SARS-CoV-2 in Vero cells. The cells were inoculated with viruses at a multiplicity of infection = 0.1. At each time point, the supernatant was collected, and the viral titer was determined using plaque assay. (**B**) Comparison of the complete sequence of wild-type SARS-CoV-2 with Vero cell-adapted SARS-CoV-2. Non-synonymous mutations are shown on each genome. Common mutations between the Kng and B-1 strains are listed in bold font. (**C**) Crystal structure of the trimeric S protein from SARS-CoV-2 (PDB accession number 7BNM). Amino acid residues 68–76, G614, H655, R685, P812, and Q853 are indicated in red, green, blue, orange, yellow, and purple circles. (**D**) Western blotting of infected cells with wild type (**P0**), adapted (**P30**) Kng, and B-1 strains at 24 h post-infection (hpi). Full-length (FL) and cleaved (**S1**) S proteins are shown under normal gain exposure and cleaved S proteins are shown under high gain exposure. (**E**) The ratio of signal density for FL compared with that for S1 was calculated using the ImageJ 1.53e software. (**F**) The infectivity of the mutated spike protein of B-1 strain using VSV-pseudotype virus. The Vero cells were infected with pseudotype viruses for 15 h. The luciferase activity was measured and has been shown as relative light units (RLUs). *0.01 < *P* < 0.05, **0.005 < *P* < 0.01, ***P < 0.005.

We generated Vero cell-adapted SARS-CoV-2 through the serial passage of two strains, SARS-CoV-2/Hu/DP/Kng/19-020 (Kng strain; LC528232) and 2020/BIKEN/B-1 (B-1 strain; LC603286), belonging to lineages B and B.1.1, respectively. Both are prototype strains isolated before the appearance of variants of concern (VOC). These two strains are distinct in the D614G mutation in the S protein, that is, the 614th amino acid of the Kng strain is aspartate, whereas that of the B-1 variant is glycine. The culture medium of Vero cells infected with SARS-CoV-2 strains was inoculated onto fresh Vero cells. Viral passaging was repeated 30 times, and the passaged and cloned Kng and B-1 strains were named Kng P30 and B-1 P30, respectively. The generated Vero cell-adapted SARS-CoV-2 strains showed a significantly increased growth in Vero cells than that exhibited by their parental strains (Fig. 1A). Genome sequences of the adapted strains were analyzed using next-generation sequencing. Twelve and four non-synonymous mutations were detected in the Kng P30 and B-1 P30 strains, respectively. A comparison of Kng P30 with Kng P0 (parental strain) resulted in the detection of the following mutations, that is, A54T at nsp3; N4D and V212L at nsp4; A32V at nsp10; K417G and K478R at nsp12; Δ68–76, V622F, H655Y, S735L, and F1089L at the S protein; and R191L at the N protein. Comparing B-1 P30 with B-1 P0, the Δ68–76, H655Y, P812L, and Q853L mutations were detected in the S protein (Table 1; Fig. 1B). Interestingly, we observed two common mutations, Δ68–76 and H655Y, in the S protein between the Kng P30 and B-1 P30 strains, introduced in early passages (Table 1). Referring to the results of cryo-EM (35), both Δ68–76 and H655Y mutations located in the S1 subunit were on the surface of the S protein (Fig. 1C). Although H655Y is close to the furin cleavage site (R685), the Δ68–76 mutation is not (Fig. 1C). Since the mutations were not close to each other, direct interactions between these mutations may be unlikely. Although the Δ68–76 mutation was rarely detected in naturally circulating strains, a similar deletion, Δ69–70, was detected in various SARS-CoV-2 strains, including the Omicron variant. The H655Y mutation has also been detected in the Omicron variant (22, 23, 31).

**TABLE 1:** Major non-synonymous mutations in Vero cell-adapted SARS-CoV-2 strains

Strain	Location	A54	N4	V212	A32	K417	K478	68–76	V622	H655	S735	P812	Q853	F1089	R191
	Gene	nsp3	nsp4	nsp4	nsp10	nsp12	nsp12	S	S	S	S	S	S	S	N
Kng P0		−^*a*^	−	−	−	−	−	−	−	−	−	−	−	−	−
Kng P13		T	D	−	−	G	R	Deletion	F	Y	−	−	−	F and L	L
Kng P20		T	D	−	−	G	R	Deletion	F	Y	−	−	−	L	L
Kng P25		T	D	L	V	G	R	Deletion	F	Y	L	−	−	L	L
Kng P30		T	D	L	V	G	R	Deletion	F	Y	L	−	−	L	L
B-1 P0		−	−	−	−	−	−	−	−	−	−	−	−	−	−
B-1 P7		−	−	−	−	−	−	Deletion	−	Y	−	−	−	−	−
B-1 P15		−	−	−	−	−	−	Deletion	−	Y	−	L	−	−	−
B-1 P20		−	−	−	−	−	−	Deletion	−	Y	−	L	Q and L	−	−
B-1 P25		−	−	−	−	−	−	Deletion	−	Y	−	L	L	−	−
B-1 P30		−	−	−	−	−	−	Deletion	−	Y	−	L	L	−	−

^*a*^ −, no mutation.

Previous reports have suggested that the Δ69–70 and H655Y mutations are associated with enhanced S1/S2 cleavage of the S protein (21, 31). Therefore, to investigate whether these mutations affect S protein cleavage, lysates from cells infected with Kng P30 and B-1 P30 were subjected to immunoblotting with an anti-S1 antibody. As expected, the adapted strains showed increased cleavage of S proteins at the S1/S2 site than in the wild-type strain, as ascertained from the levels of full-length and cleaved S proteins (Fig. 1D and E). To further determine the role of mutations in enhanced viral growth, the infectivity of the mutated S protein was evaluated using VSV-based pseudotype viruses covered with the S protein. While the Δ68–76 mutation decreased the viral infectivity, H655Y mutations showed enhanced viral infectivity (Fig. 1F). The combination of Δ68–76 and H655Y significantly enhanced infectivity, indicating that the two mutations function synergistically (Fig. 1F). P812L and Q853L mutations might not influence the enhanced infectivity induced by Δ68–76 and H655Y mutations (Fig. 1F).

### Pathogenicity of Vero cell-adapted SARS-CoV-2 strains

**Fig 2:**
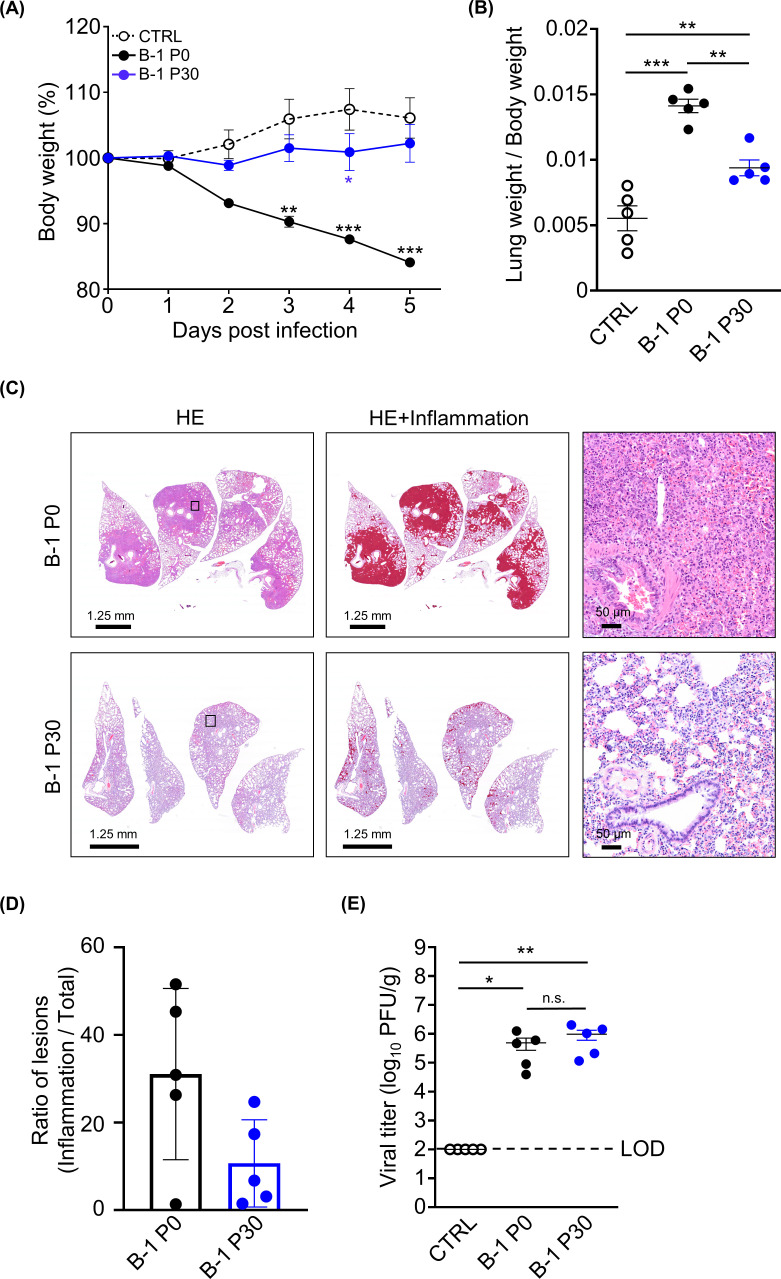
Virus challenge experiment involving infection with Vero cell-adapted SARS-CoV-2 in hamster models. (**A**) The body weights of hamsters infected or uninfected with SARS-CoV-2 and Vero cell-adapted SARS-CoV-2 (*n* = 5). (**B**) The ratio of lung weight to body weight of hamsters infected with SARS-CoV-2. (**C**) The histopathological images of hamster lungs infected or uninfected with SARS-CoV-2. The images display sections stained with hematoxylin and eosin (HE) (left). The images highlighting the inflammation are shown (middle). The squares show the magnified area in the images (right). The scale bars are shown on the left below. (**D**) The ratio of the area exposed to lesions in the lung to the total lung area of infected hamsters was calculated by comparing the area of inflammation with the total lung area. (**E**) The viral titers of lungs. The viral titers were determined using plaque assay. The limit of detection is indicated as a broken line. *0.01 < *P* < 0.05, **0.005 < *P* < 0.01, ***P < 0.005.

Herein, we used the B-1 strain for subsequent analyses unless otherwise stated, as almost all circulating SARS-CoV-2 strains have a D614G mutation, the B-1 strain was relatively more virulent than the Kng strain in hamsters (data not shown), and B-1 P30 was mutated at only four points in the S protein. Although Vero cell-adapted SARS-CoV-2 grows well and enhances the cleavage of the S protein *in vitro*, its pathogenicity *in vivo* remains unknown. To clarify the pathogenicity of the mutations of the S protein, we intranasally inoculated Vero cell-adapted SARS-CoV-2 (B-1 P30) into hamsters and observed its progression. Infection with the B-1 P0 strain resulted in lower body weights in infected hamsters compared with the body weights of hamsters infected with the B-1 P30 strain (Fig. 2A). In addition, the ratio of lung weight/body weight (lung inflammation indicator) of the B-1 P30 group was significantly lower than that of the B-1 P0 group (Fig. 2B). The histopathological analysis also supported the milder inflammation observed in B-1 P30-infected lung than in B-1 P0-infected lung, although the difference was not statistically significant (Fig. 2C and D). B-1 P0 was relatively more virulent than the B-1 P30 assessed in terms of body weight loss and lung inflammation; yet, there was no significant difference in the viral titer in the lungs (Fig. 2E). Together, these data imply that the pathogenicity of B-1 P30 is attenuated in the hamster model, at least in terms of body weight loss and lung inflammation.

### Growth of recombinant SARS-CoV-2 strains bearing single or multiple mutations in their S proteins

**Fig 3:**
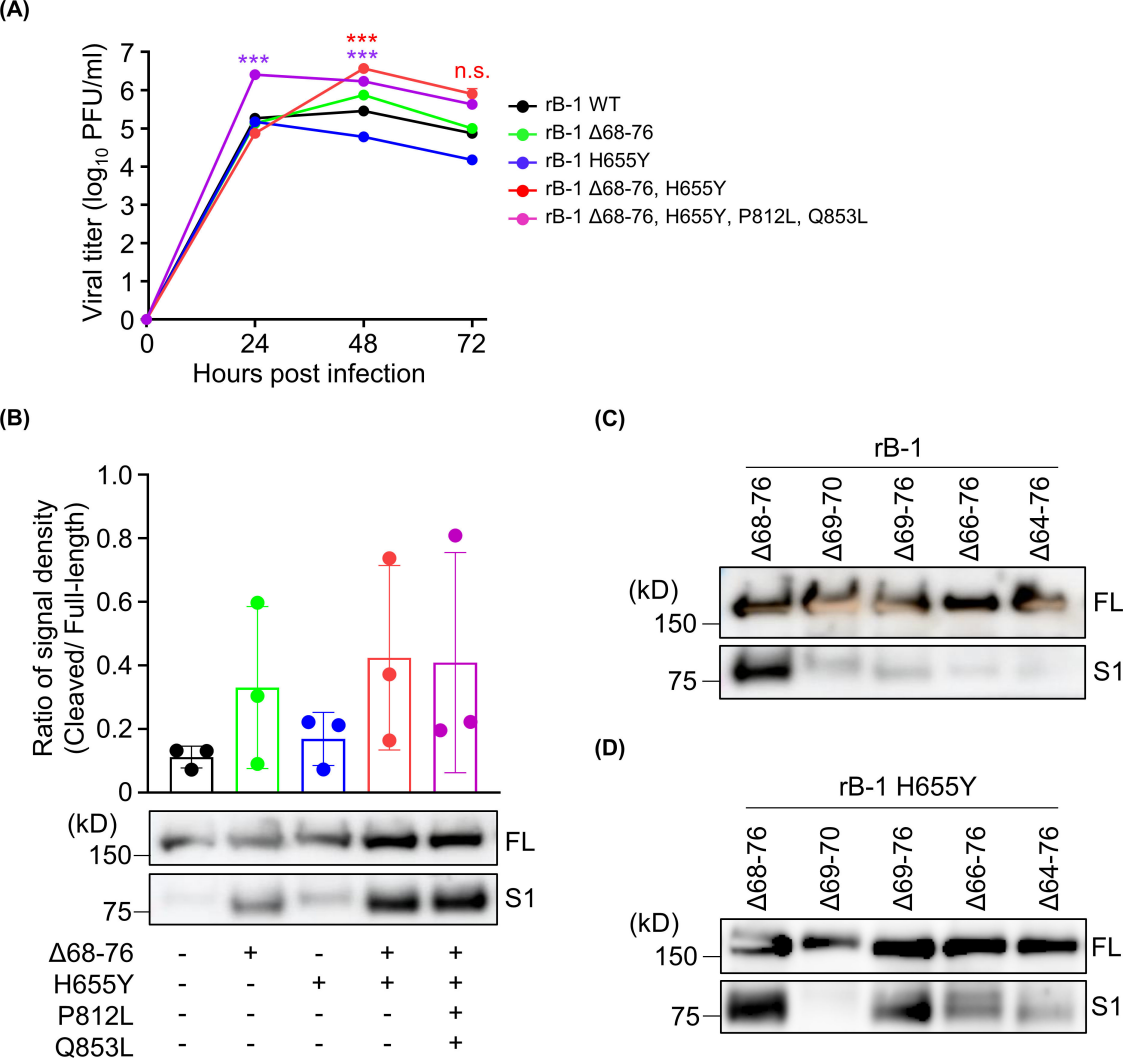
Virological analysis of recombinant SARS-CoV-2 strains bearing single or multiple mutations. (**A**) Growth kinetics of recombinant viruses derived using the CPER method. Vero cells were plated onto a 24-well plate and inoculated with each virus strain at a multiplicity of infection = 0.1. The viral titer was determined using plaque assay. (**B**) Western blotting of infected cells with B-1-based recombinant SARS-CoV-2. The cellular levels of full-length (FL) and cleaved (**S1**) spike proteins are shown. The signal density of spike proteins of recombinant SARS-CoV-2 strains was calculated using the ImageJ 1.53e software. (**C, D**) Western blotting of infected cells with B-1-based recombinant SARS-CoV-2 bearing partial deletion in the NTD (**C**) or partial NTD deletion and H655Y mutation (**D**). The levels of FL and cleaved (**S1**) spike proteins are shown. ***P < 0.005.

We generated recombinant SARS-CoV-2 strains mutated at single or multiple points through circular polymerase extension reaction (CPER) (36) to identify amino acid residues responsible for enhanced viral growth and attenuated pathogenesis and examine the virological characteristics of the mutations. The recombinant viruses bearing the Δ68–76 and H655Y mutations revealed significantly increased levels of growth over the wild-type strain (Fig. 3A). The viruses carrying the additional mutations P812L and Q853L, the same as those in B-1 P30, also showed significant levels of growth and seemed to reach the peak in growth faster than in viruses bearing only the Δ68–76 and H655Y mutations (Fig. 3A). The viruses carrying single-point mutations (Δ68–76 or H655Y) did not show a significant increase in the viral growth (Fig. 3A). These results suggest that the two mutations Δ68–76 and H655Y in the S protein are associated with enhanced viral growth.

### Degree of cleavage of the S proteins in strains bearing the combination of the Δ68-76 and H655Y mutations

The cleavage patterns of the S proteins of the recombinant viruses based on the B-1 strain were visualized using western blotting to determine the amino acid residues responsible for the cleavage of the S proteins. Cleavage of the S protein was enhanced in cells infected with the virus carrying the Δ68–76 and H655Y mutations and in cells infected with viruses carrying either the Δ68–76 or H655Y mutations alone (Fig. 3B). These results also coincide with reports that used pseudotype virus (21, 31). The combination of Δ68–76 and H655Y markedly enhanced the cleavage. It has been suggested that enhancement in S protein cleavage is closely related to enhanced viral growth. Enhanced cleavage was also analyzed using the Kng strain-based recombinant viruses. An anti-S2 antibody was used to determine which sites (S1/S2 or S2′ or both) were cleaved. The results confirmed that the Δ68–76 and H655Y mutations contributed to the efficient cleavage of the S protein in the Kng strain (Fig. S1A). However, the S2′ site remained undetected, indicating enhanced S protein cleavage at the S1/S2 site (Fig. S1B).

Next, we compared the Vero cell-adapted SARS-CoV-2 with other wild-type strains. Hitherto, large amounts of SARS-CoV-2 genome data have been deposited in the GISAID (https://www.gisaid.org/). It has been reported that SARS-CoV-2 strains carrying various types of partial NTD deletions (i.e., Δ69–70, Δ69–76, Δ66–76, and Δ64–76) with or without the H655Y mutation are currently circulating, of which the Δ69–70 mutation with H655Y is the most prevalent. However, the combination of the Δ68–76 and H655Y mutations has not yet been reported in naturally occurring strains. To investigate the virological role of the interaction between the partial NTD deletion and H655Y, we generated recombinant SARS-CoV-2 with various partial deletions of the NTD (and H655Y) using the CPER method. The S protein with the Δ68–76 mutation was cleaved most effectively among the recombinant NTD-partially deleted SARS-CoV-2 strains (Fig. 3C). The effect of the partial NTD deletion was analyzed in the presence of the H655Y mutation. The H655Y mutation increased the cleavage of the S protein when the partial NTD deletion was concomitantly introduced (Fig. 3C and D). Notably, the enhancement in S protein cleavage was more effective in strains carrying the Δ68–76 and H655Y mutations than in strains carrying the Δ69–70 and H655Y mutations, as commonly detected in the Omicron variant. It was indicated that Δ68–76 was relatively more suitable for enhancement in cleavage than the other mutations. The combination of H655Y and NTD synergistically enhanced S protein cleavage.

### Dependence of the recombinant SARS-CoV-2 strains on host proteases in infecting Vero cells

**Fig 4:**
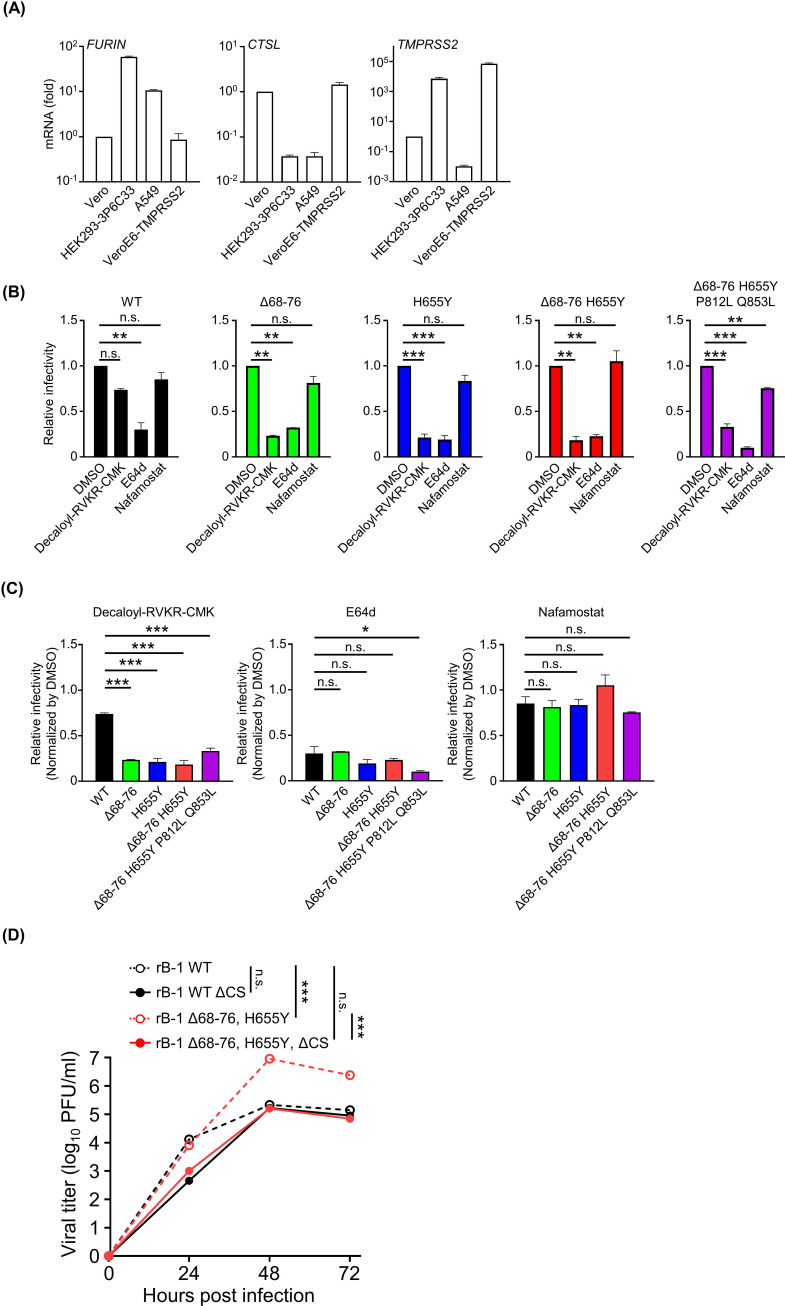
Dependence of recombinant SARS-CoV-2 strains on host proteases for entry and the infection. (**A**) The expression levels of the host factors according to the entry of SARS-CoV-2. Cellular mRNAs were extracted from each cell line and quantified using real-time qRT-PCR. The value of Vero cells was normalized as 1.0. (**B**) The infectivities of recombinant SARS-CoV-2 strains in the presence of protease inhibitors. Vero cells were treated with the inhibitors for 1 h and infected with the virus for 20 h. The infected cells were visualized by indirect fluorescent assay and counted. (**C**) The summarized data using each inhibitor were shown. The ratios of the infectivities were normalized based on the results obtained under DMSO treatment. (**D**) Growth kinetics of the furin cleavage site deleted (ΔCS) recombinant viruses derived using CPER. Vero cells were plated in a 24-well plate and inoculated with each virus at a multiplicity of infection of 0.1. The viral titer was determined using a plaque assay. *0.01 < *P* < 0.05, **0.005 < *P* < 0.01, ***P < 0.005.

To determine the host proteases involved in the enhanced cleavage, the inhibitory assay using protease inhibitors was carried out. First, the mRNA levels of host proteases in Vero cells were determined using real-time quantitative reverse transcription-polymerase chain reaction (qRT-PCR). Although CTSL revealed higher levels of expression in Vero cells than in HEK293-3P6C33 and A549 cells, furin and TMPRSS2 were expressed at low levels in Vero cells (Fig. 4A). Vero cells treated with decanoyl-Arg-Val-Lys-Arg-chloromethylketone (decanoyl-RVKR-CMK, furin inhibitor), E64d (CTSL inhibitor), or nafamostat (TMPRSS2 inhibitor) were challenged with the virus to test their susceptibility to recombinant SARS-CoV-2 strains. All viruses were generally inhibited by E64d and not inhibited by nafamostat. There were no significant differences between the wild-type strain and recombinant viruses with S protein mutations, except for the combination of E64d and virus with Δ68–76, H655Y, P812L, and Q853L (Fig. 4B and C). However, recombinant viruses bearing any mutation were relatively more sensitive to decanoyl-RVKR-CMK than the wild-type virus. These results indicate that the enhanced entry of adapted SARS-CoV-2 into Vero cells was mainly related to furin-dependent cleavage of the S protein.

The cleavage of the S protein processed by furin was most likely responsible for the enhanced viral growth. We generated a B-1-based recombinant SARS-CoV-2 strain with abolished furin cleavage site (ΔCS) by converting the amino acid sequence of the multibasic region “TNSPRRA” into “SLL,” which is the sequence of SARS-CoV strain to confirm the relationship between the utilization of furin and viral growth (4). The enhancement of viral growth by the Δ68–76 and H655Y mutations was lost by an additional inserted mutation, ΔCS, validating that cleavage of the S protein by furin was responsible for enhanced viral growth (Fig. 4D). Loss of the enhanced cleavage at the S1/S2 site in the recombinant SARS-CoV-2 strains was confirmed by western blotting (Fig. S2). Altogether, the results for the recombinant viral strains showed that Δ68–76 and H655Y increased viral growth by cleaving the S protein in a furin-dependent manner. These data suggest that furin-mediated cleavage of the S protein correlates positively with virus growth in Vero cells.

### Pathogenicity of recombinant SARS-CoV-2 strains

**Fig 5:**
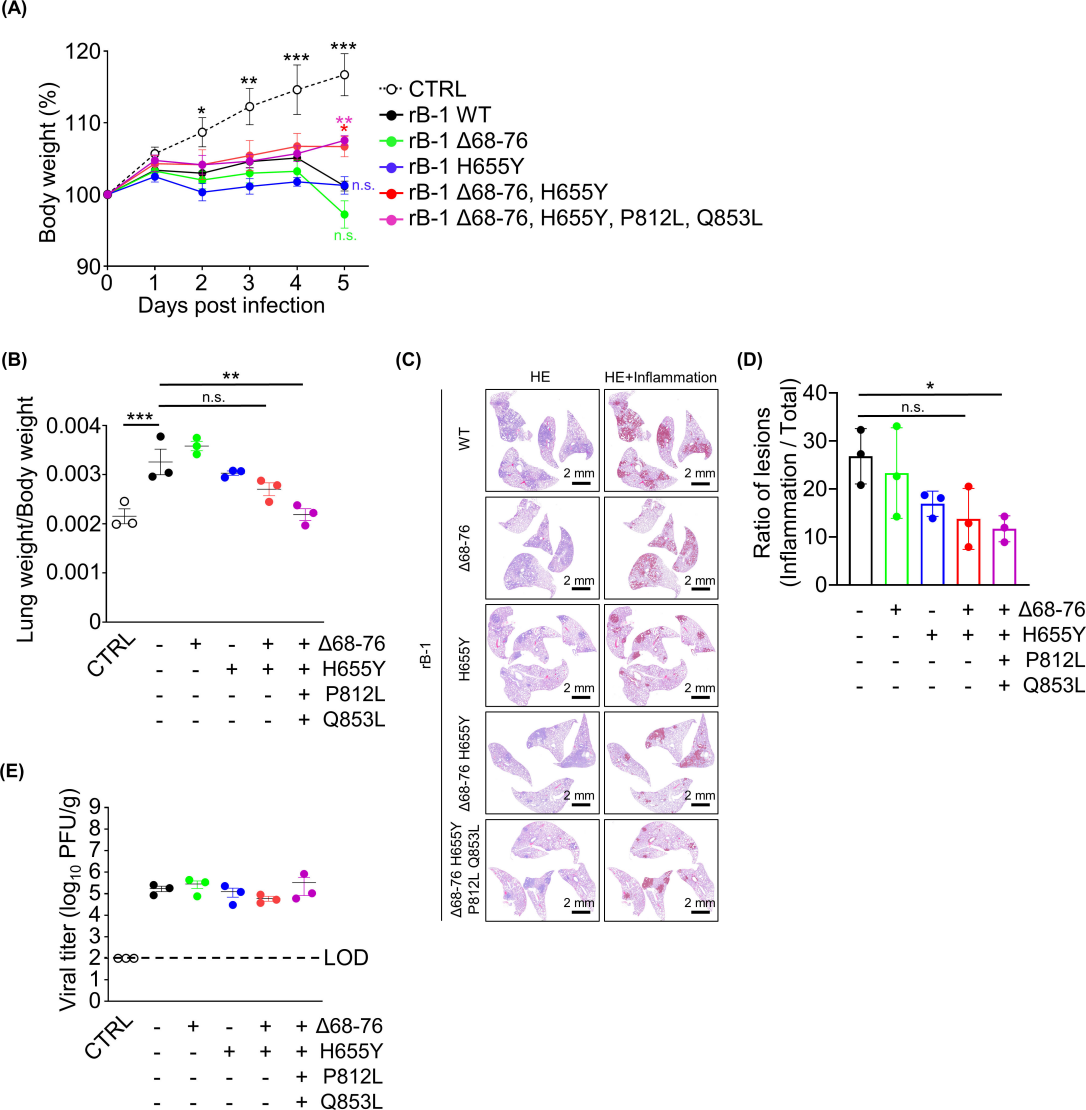
Pathogenicity of recombinant viruses bearing single or multiple mutations *in vivo*. (**A**) The body weights of hamsters infected or uninfected with recombinant SARS-CoV-2 strains were generated using the CPER technique (*n* = 3). (**B**) The ratios of lung weight to body weight of hamsters infected with the SARS-CoV-2 strains. (**C**) The histopathological images of the lungs of hamsters infected or uninfected with recombinant SARS-CoV-2 strains. Images displaying tissue sections stained with hematoxylin and eosin (HE) are shown on the left. Images highlighting tissue inflammation are shown on the right. The scale bars are shown on the left below. (**D**) The ratios of lesions in the lungs of infected hamsters were calculated by comparing the area under inflammation with the total lung area. (**E**) The viral titers of lungs. The viral titers were determined using plaque assay. The limit of detection is indicated as a broken line. *0.01 < *P* < 0.05, **0.005 < *P* < 0.01, ***P < 0.005.

The present study revealed that the Δ68–76 and H655Y mutations were responsible for enhanced cleavage in the Kng P30 and B-1 P30 strains *in vitro*. However, the effects of these mutations on pathogenicity remain unclear. Furthermore, the mutation that causes the attenuation of B-1 P30 remains unclear. Hamsters were intranasally inoculated with recombinant SARS-CoV-2, followed by an examination of the body weights and condition of the lungs to identify the amino acids responsible for attenuating the pathogenicity of B-1 P30. Recombinant SARS-CoV-2 with the S protein of the wild-type strain only mildly affected body weight compared with the wild-type strain (Fig. 2A and 5A).

Previous studies have reported the attenuated pathogenicity and replication of recombinant viruses compared with the parental viruses (36–38), consistent with our findings. A recombinant virus generated by a reverse genetic system may show mild pathogenicity due to a highly cloned viral population. Recombinant SARS-CoV-2 with the S protein of the B-1 P30 strain (Δ68–76, H655Y, P812L, and Q853L) and the double mutation (Δ68–76 and H655Y) caused milder body weight loss than that caused by the wild-type virus at 5 days post-infection (dpi). The other single-point mutant recombinant SARS-CoV-2 strains showed no significant differences (Fig. 5A). Lung inflammation was milder in the recombinant B-1 P30 group than in the wild-type group (Fig. 5B). Histopathological analysis of the infected lungs supported mild inflammation in the recombinant B-1 P30 and double mutation groups (Fig. 5C and D). However, no significant differences were observed between the double mutation and wild-type groups, although the former showed a tendency for mild inflammation (Fig. 5B through D). The other recombinant strains showed a pathogenicity similar to the pathogenicity of the wild-type virus (Fig. 5B through E). There was no difference in viral titers among different groups despite improvements in body weight loss and lung inflammation in mutant strains (Fig. 5E). Taken together, the combination of two or four mutations in the S protein, but not a single mutation, may be involved in alleviating the adverse effects of the SARS-CoV-2 B-1 strain *in vivo* in terms of body weight and lung inflammation.

### Fusion activity of Vero cell-adapted SARS-CoV-2

Fusion activity is a crucial factor in the pathogenicity (39–41). To understand the attenuation caused by the mutations, we focused on the fusion activity of the S protein of the B-1 P30 strain. VeroE6-TMPRSS2 cells were transfected with plasmids expressing the mutated S protein, including D614G. Fusion activity was increased by the H655Y mutation but not by the Δ68–76 deletion (Fig. S3). Together, these two mutations resulted in a fusion activity comparable to that of H655Y alone. However, four simultaneous mutations (Δ68–76, H655Y, P812L, and Q853L) detected in the B-1 P30 strain did not show enhanced fusion activity (Fig. S3). These results suggest that the P812L and Q853L mutations are associated with decreased fusion activity, contributing to the attenuation of pathogenicity.

### Evaluation of the Vero cell-adapted SARS-CoV-2 strain as an inactivated vaccine

**Fig 6:**
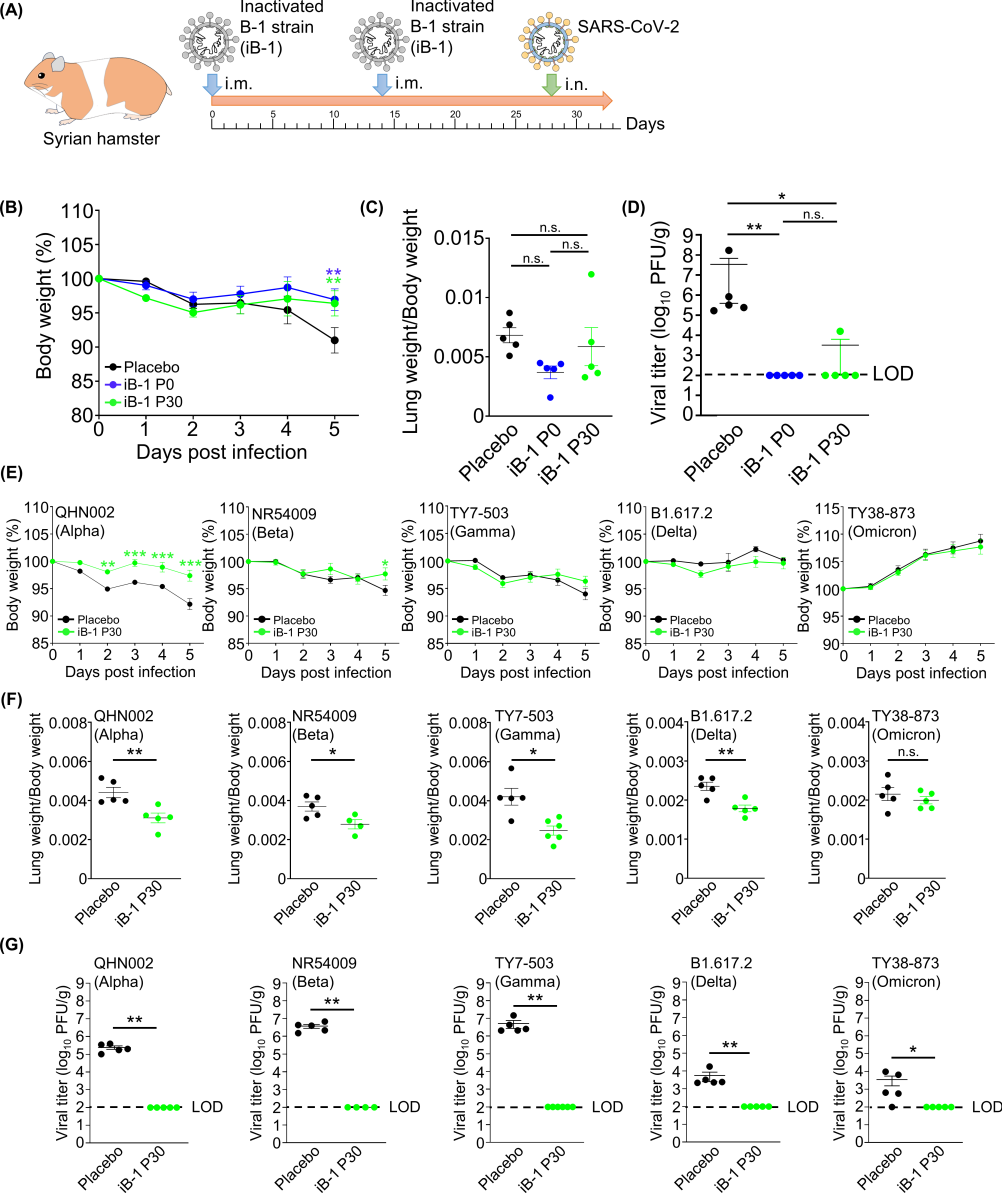
Vaccination of hamsters using inactivated Vero cell-adapted SARS-CoV-2 strains. (**A**) Schematic diagram of the vaccination of hamsters using the inactivated SARS-CoV-2 strains and the inoculation of SARS-CoV-2 in the hamsters. (**B**) The body weights of immunized hamsters after challenge with the B-1 P0 strain. (**C**) The ratios of lung weight to body weight of hamsters infected with the B-1 P0 strain. (**D**) The viral titers of hamster lungs infected with the B-1 P0 strain. The viral titers were determined using plaque assay. The limit of detection (LOD) is indicated as a broken line. (**E**) The body weights of immunized hamsters after challenge with SARS-CoV-2 variants. (**F**) The ratios of lung weight to body weights of hamsters infected with SARS-CoV-2 variants. (**G**) The viral titers in hamster lungs infected with SARS-CoV-2 variants. The viral titers were determined using plaque assay. LOD is indicated as a broken line. *0.01 < *P* < 0.05, **0.005 < *P* < 0.01, ***P < 0.005.

The established Vero cell-adapted SARS-CoV-2 grew well in Vero cells, a cell line approved for vaccine production (Fig. 1A). A primary advantage of using a highly productive virus for vaccine production is cost reduction in manufacturing. Moreover, the pathogenicity of the adapted SARS-CoV-2 was lower than that of the wild type (Fig. 2A through D), which contributed to a decrease in the handling risk. Therefore, we evaluated the immunogenicity of Vero cell-adapted SARS-CoV-2 as an inactivated vaccine. The viruses were inactivated using β-propiolactone and intramuscularly inoculated into hamsters twice at 2-week intervals. After immunization, the hamsters were administered intranasally with the parental B-1 P0 strain (Fig. 6A). The hamsters immunized with the inactivated B-1 P0 and B-1 P30 strains (iB-1 P0 and iB-1 P30, respectively) showed significantly reduced body weight loss than in the placebo group at 5 dpi (Fig. 6B). Unexpectedly, the ratio of lung weight/body weight in hamsters, indicating lung inflammation did not significantly differ between the placebo and iB-1 groups, although the immunized groups showed a tendency for milder inflammation (Fig. 6C). However, the histopathological analysis showed reduced inflammation in the immunized groups (Fig. S4). Notably, the infectious virus titer in the lungs decreased significantly in the iB-1 group (Fig. 6D), further confirmed by immunohistochemical staining with an anti-N antibody (Fig. S4). There was no difference between groups immunized with iB-1 P0 and iB-1 P30 strains in body weight, lung inflammation, or viral titer (Fig. 6B through D), which indicates that iB-1 P30 is as effective as iB-1 P0 and that immunogenicity may be conserved among the B-1 P0 and B-1 P30 strains.

To further evaluate the protective efficacy of the inactivated vaccine, other viral strains, including Alpha, Beta, Gamma, Delta, and Omicron, were used in the challenge experiment (Fig. 6E through G). The body weights of hamsters infected with the alpha strain decreased, and this decrease was prevented by immunization with iB-1 P30. However, it was difficult to control body weight loss in hamsters infected with the other strains (Fig. 6E). The iB-1 P30-immunized hamsters infected with the Alpha, Beta, Gamma, and Delta strains showed decreased lung inflammation (Fig. 6F). The ratio of lung weight/body weight in hamsters infected with the Omicron strain was not different between the placebo and iB-1 P30 groups (Fig. 6F). As small limited lesions were observed in the lungs of hamsters infected with the Omicron strain during sample collection (data not shown), evaluating its protective activity may be difficult. Notably, the infectious virus could not be detected in the lungs of the iB-1 P30-immunized hamsters in any group (Fig. 6G).

**Fig 7:**
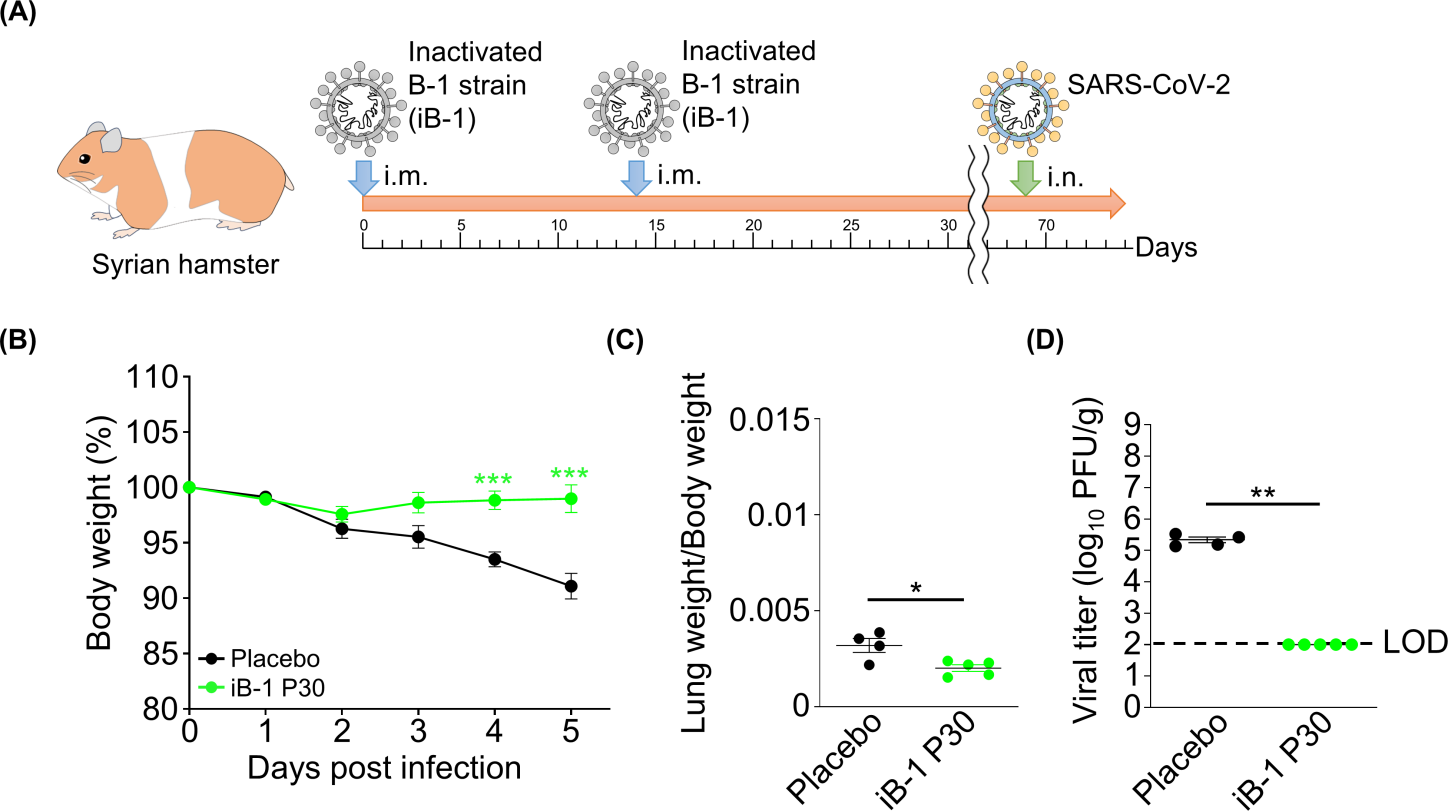
Long-term trial in vaccinated hamsters using inactivated Vero cell-adapted SARS-CoV-2 strains. (**A**) Schematic diagram of vaccination with inactivated SARS-CoV-2 strains and inoculation of SARS-CoV-2 in hamsters. (**B**) Body weights of immunized hamsters after B-1 P0 challenge. (**C**) The ratios of lung weight to body weight in hamsters infected with the B-1 P0 strain. (**D**) Viral titers in hamster lungs infected with the B-1 P0 strain. Viral titers were determined using plaque assay. The limit of detection is indicated by a broken line. *0.01 < *P* < 0.05, **0.005 < *P* < 0.01, ***P < 0.005.

Finally, a long-term vaccination trial was conducted. At 69 days after the last immunization, the immunized hamsters were infected with the B-1 P0 strain (Fig. 7A). Immunization protected the infected hamsters from weight loss and lung inflammation (Fig. 7B and C). Infective SARS-CoV-2 was not detected in the lung (Fig. 7D), which indicated that the immune response may still be sufficiently active to prevent the infection of SARS-CoV-2. Immunization with iB-1 P30 effectively protected hamsters from SARS-CoV-2 infection for at least 2 months after this vaccination.

## DISCUSSION

In this study, we established a Vero cell-adapted SARS-CoV-2 strain with high viral growth. Vero cells support the replication of various viruses as they are deficient in type I interferon production (42). This susceptibility to viruses can help to elucidate the mechanism underlying viral infection and also help develop highly productive vaccine strains through adaptation (43–47). For adaptation, we chose two different SARS-CoV-2 strain types, that is, the Wuhan and the Europe strain types. These two strains are distinct in the D614G mutation in the S protein, that is, the 614th amino acid of the Wuhan/Kng strain is aspartate, whereas that of the Europe/ B-1 strain is glycine. The D614G mutation alters the conformation of the S protein keeping the RBD “up,” increasing viral transmission and infectivity (48–50). The B-1 P30 strain possesses mutations only in the gene coding for the S protein, whereas the Kng P30 strain possesses mutations in the gene coding for the S protein and other viral protein genes. Mutations in other genes of Kng P30 possibly support the enhancement of viral growth, which warrants further investigation.

The S proteins of Kng P30 and B-1 P30 strains have two common mutations, Δ68–76 and H655Y, which contributed to effective S1/S2 cleavage and viral growth enhancement. Specifically, the Δ68–76 mutation enhanced S protein cleavage at the S1/S2 site relatively more strongly than that caused by a similar previously reported deletion, that is, Δ69–70 (21). Although the Δ68–76 or H655Y mutations enhanced cleavage at the S1/S2 site individually, the growth of the recombinant SARS-CoV-2 showed no enhancement, indicating that these mutations may not be individually sufficient to improve viral growth. These two mutations synergistically enhanced cleavage at the S1/S2 site and viral growth. Notably, the other partial NTD deletions (Δ69–70, Δ69–76, Δ66–76, and Δ64–76) did not show a robust enhancement in cleavage when present alone, and the H655Y mutation enhanced the extent of S protein cleavage caused by partial NTD deletions except that caused by Δ69–70. The protein region bearing the 69–76 amino acids at the NTD comprises the loop structure, and the Δ69–70 mutation reportedly pulls the NTD structure inward (21). Other partial NTD deletions, for example, Δ68–76, may be involved in altering the conformation of the NTD structure, which, in turn, causes altered virological characteristics. However, the mechanism underlying the enhanced cleavage caused by these mutations and the synergistic effect of H655Y and partial NTD deletions is still unknown and warrants further studies. Omicron strain (lineage B.1.1.529) bearing the Δ69–70 and H655Y mutations is a primary VOC, causing the COVID-19 pandemic worldwide. Considering that the Δ68–76 deletion showed higher efficacy of S protein cleavage at the S1/S2 site than that exhibited by the Δ69–70 deletion, the SARS-CoV-2 variants harboring a combination of Δ68–76 and H655Y are possibly the dominant variants, causing a pandemic. Hitherto, a several times passaged SARS-CoV-2 strain (lineage B.1) in Caco-2 cells also harbored Δ68–76 without H655Y (51), while the wild-type strains mutated Δ68–76 without H655Y were detected in Taiwan (52) and Pakistan (53). Further epidemiological studies remain warranted to monitor the emergence of variants harboring Δ68–76 and H655Y.

Currently, H655Y is considered a crucial mutation owing to several studies reporting on the acquisition of the H655Y mutation. Serial passage of a recombinant VSV expressing SARS-CoV-2 S protein in Vero cells led to the H655Y mutation in the recombinant virus (30). Another study reported that during viral passages of SARS-CoV-2, in the presence of an antibody cocktail, in Vero cells, the virus acquired a mutation in H655Y (54). Moreover, the mutation was introduced because of the transmission of the virus to experimental animals (32, 33). Although the role of H655Y *in vivo* remains unknown, the enhanced cleavage or the enhanced cell-cell fusion might cause effective viral transmission. Our data, along with those of previous studies, indicate the importance of H655Y for viral fitness and adaptation. The H655Y mutation in the pseudotype virus reportedly enhances cleavage at the S1/S2 site and cell-cell fusion, substantiating our results using recombinant viruses. Notably, the enhancement in the fusion activity of strains bearing H655Y was suppressed by P812L and Q853L mutations; these amino acid residues are located near the FP and are closely associated with cell fusion (55). The mutations may reduce cell-membrane fusion. In addition, we showed that the replication kinetics of the recombinant virus bearing H655Y was similar to that of the wild-type virus, indicating that cell fusion activity is not related to enhanced viral growth in Vero cells, wherein TMPRSS2 is expressed at low levels. Examination of viral kinetics in other cell lines is of interest.

Generally, the wild-type SARS-CoV-2 enters Vero cells *via* the CTSL-dependent endocytosis route (11). It has been reported that SARS-CoV-2 adapted to VeroE6 cells use CTSL relatively more effectively than other proteases (27). Another report showed that the Δ69–70 deletion in the pseudotype virus does not enhance the dependency on CTSL for virus entry (21), consistent with our findings. The susceptibility of the recombinant B-1 P30 strain for the inhibitor of CTSL significantly became higher than the wild-type virus, indicating the recombinant B-1 P30 strain mainly used the endocytosis route processed by CTSL. However, its dependency on CTSL for entering the host cells failed to show a dramatic enhancement. Recombinant B-1 P30 was relatively more susceptible to furin inhibitors than wild-type, indicating that the adapted viruses evolved to effectively utilize furin, not sufficiently used for entering into Vero cells. This result is the opposite of previous findings on the mutations that abolish the furin cleavage site in the Vero cell-adapted SARS-CoV-2 strain (26–28). Most previous studies used VeroE6 cells which had slightly different characteristics compared with Vero cells. Especially, the TMPRSS2 expression level in VeroE6 cells was lower than in Vero cells (9). The balance of the expression levels of host proteases might be important for the evolution of SARS-CoV-2. Although the exact reason for the induction of these contradictory mutations remains unclear, a difference in the history of passage through Vero cells or the expression pattern of host proteases including furin may cause this difference.

The B-1 P30 strain was attenuated in hamsters. Although the recombinant strain with the Δ68–76 and H655Y mutations also underwent attenuation, the recombinant strain possessing additional mutations, P812L and Q853L, was relatively more attenuated. In a previous report, the SARS-CoV-2 variant carrying a deletion of the furin cleavage site did not affect the body weights of hamsters and mice (18). In a ferret model, the furin cleavage site is essential for transmission between ferrets (19). The QTQTN motif upstream of the furin cleavage site also plays a role in the effective cleavage at the S1/S2 site and viral attenuation (56). The loss of furin-mediated cleavage induces dramatic viral attenuation. However, our results suggested a possibility that the excessive cleavage of the S protein would also decrease pathogenicity. For effective infection, the S protein may be appropriately cleaved.

To reveal the mechanism underlying attenuation, we analyzed another critical role of the S protein, that is, cell-cell fusion. The Delta strain, which has high fusion activity due to the P681R mutation, causes severe weight loss in hamsters (57). Although the S protein bearing Δ68–76 and H655Y showed high fusion activity, the S protein possessing additional mutations, that is, P812L and Q853L did not. The reduction of the cell-cell fusion by P812L and Q853L mutations might cause the reduction of cell damage. The mutations did not affect viral replication but might reduce the damage to the lung *in vivo*. It is hypothesized that the co-existence of these four mutations in the adapted SARS-CoV-2 strain (B-1 P30) causes attenuation due to increased cleavage activity and decreased cell fusion activity *in vivo*. Although we could not determine the mutations critical for attenuation, these four mutations contributed to the attenuation of viral pathogenicity. The relationship between these complex viral characteristics and host immune responses warrants further assessment.

Due to the essential role of the S protein in viral infections, vaccines targeting only the S protein (e.g., mRNA and viral vector vaccines) are effective and are widely used (58–60). However, the S protein is frequently mutated to escape neutralizing antibodies. Immunization with the current vaccine may not protect against infection by the emerging SARS-CoV-2 variants. Indeed, the antigenicity of the S protein of the Omicron strain differs from that of the prototype strains (61). The effect of mRNA vaccines that encode the prototype S protein as an immunogen is weakened against the Omicron strain (62, 63). By contrast, inactivated vaccines target all viral structural proteins. The inactivated vaccines induce antibodies against the S protein and also against other antigenic viral proteins, including M and N. As the M and N proteins are relatively conserved among different variants, the inactivated vaccines may induce immunity against the new emerging SARS-CoV-2 variants. In a previous study, the conformation of the RBD “up” influenced by the furin cleavage elicited the neutralizing antibody. The Omicron strain remains the conformation of the RBD “down,” thereby escaping neutralization (64). The adapted strain might keep the conformation of the RBD “up,” eliciting a strongly neutralizing antibody. Our results showed that the immunogenicity of the adapted strain was similar to that of the wild-type strain. Notably, vaccination with the inactivated-adapted strain protected hamsters against infection from SARS-CoV-2 variants, including Omicron, suggesting the potency of the adapted virus as an inactivated vaccine. In addition, the adapted SARS-CoV-2 strains grow better than the wild-type strain in Vero cells, reducing the cost of the inactivated vaccines. Considering their immunogenicity and high productivity, the inactivated-adapted SARS-CoV-2 are promising candidates for vaccine development.

In conclusion, we established Vero cell-adapted SARS-CoV-2 strains that exhibited high viral growth, presumably due to furin-mediated enhanced cleavage at the S1/S2 site. In contrast to the high growth rate observed *in vitro*, the adapted virus showed reduced pathogenicity in a hamster model. Recombinant viruses showed that a combination of two mutations, Δ68–76 and H655Y, contributed to these phenotypes. In addition, we demonstrated that the adapted virus can be used as an inactivated vaccine. Our findings contribute to elucidating the mechanisms underlying SARS-CoV-2 infection and pathogenicity to control COVID-19 and to develop an effective and stable vaccine.

## MATERIALS AND METHODS

### Cells

African green monkey kidney cells (Vero) (ATCC CCL-81), human kidney cell line 293T (ATCC CRL-3216), and human lung cell line A549 (ATCC CCL-185) were cultured using the Dulbecco’s Modified Eagle’s Medium (DMEM; Nacalai Tesque, Kyoto, Japan) containing 5% or 10% fetal bovine serum (FBS; Thermo Fisher Scientific, MA, USA), 100 U/mL penicillin and 100 µg/mL streptomycin (Nacalai Tesque). VeroE6-TMPRSS2 cells temporally expressing TMPRSS2 were purchased from the Japanese Collection of Research Bioresources Cell Bank (JCRB1819) and cultured with DMEM containing 5% FBS, 100 U/mL penicillin, 100 µg/mL streptomycin, and 1 mg/mL G418 (Nacalai Tesque). The HEK293-3P6C33 cells, which can exogenously express ACE2 and TMPRSS2 on adding tetracycline, were cultured in DMEM containing 10% FBS and 10 mg/mL blasticidin (InvivoGen, CA, USA). The culture medium was replaced with a fresh medium containing 1 mg/mL doxycycline hydrochloride (Sigma-Aldrich, MO, USA) to express ACE2 and TMPRSS2 in HEK293-3P6C33 cells (36).

### Viruses

SARS-CoV-2/Hu/DP/Kng/19-020 (Kng strain; LC528232, kindly provided by Dr. Tomohiko Takasaki and Dr. Jun-Ichi Sakuragi at the Kanagawa Prefectural Institute of Public Health) and 2020/BIKEN/B-1 (B-1 strain; LC603286) strains were propagated in Vero cells. Vero cell-adapted SARS-CoV-2 was established 30 times in the Vero cells. Briefly, Vero cells cultured in a 12-well plate at a cell density of 2 × 10^5^ cells/well (Corning, NY, USA) were challenged with the wild-type strain at a multiplicity of infection (MOI) of 0.1. At 3 dpi, the culture solution was centrifuged at 1,600 × *g* for 5 min, and the supernatant was collected. Then, 1 µL of supernatant was inoculated onto new fresh Vero cells, repeated 30 times. After 30 passages, viruses were cloned *via* plaque isolation. The major amino acid mutations in the cloned strain were confirmed by the Sanger sequencing using specific primers. The cloned and isolated SARS-CoV-2 strains were named the Kng P30 and B-1 P30 strains, respectively. The QHN002 (Alpha), NR54009 (Beta), TY7-803 (Gamma), B1.617.2 (Delta), and TY38-873 (Omicron) strains were kindly provided by the National Institute of Infectious Diseases and propagated using Vero cells. All viruses were handled at Biosafety Level 3 at Osaka University.

### Whole-genome sequencing of SARS-CoV-2

The sequence libraries were prepared using the SureSelect XT Low Input Kit (Agilent Technologies, CA, USA) and a custom panel designed against the SARS-CoV-2 reference genome (NC_045512.2) with a 6 × tiling density using a 20 bp sliding window (65). Paired-end sequencing of 2 × 100 bp reads was performed using the DNBSEQ-G400RS sequencer (MGI, Shenzhen, China). After trimming the adapter sequences using Cutadapt version 3.2, the trimmed sequence reads were aligned to the reference genome of SARS-CoV-2 (GenBank accession number: NC_045512.2) using BWA version 0.7.17. After marking duplicate reads in the BAM files using SAMtools version 1.11 and Picard in GATK 4.2.0.0, variant calling was executed using Mutect2 in GATK 4.2.0.0. The consensus sequences were obtained using BCFtools version 1.9.

### Pseudotype virus

The HEK293T cells were seated in a six-well plate at a cell density of 5 × 10^5^ cells/well (Corning). The cells were transfected with 1.0 µg of the plasmid encoding the S protein codon-optimized for expression in mammalian cell lines under the control of the CAG promoter using the TransIT-LT1 transfection reagent (Mirus, WI, USA). The medium was changed 6 h after transfection. A day after transfection, the cells were infected with VSVΔG-Luc reporter virus, a pseudotype VSV with the receptor-binding G protein replaced by the luciferase reporter, at an MOI of 0.1 and incubated at 37°C for 2 h. After incubation, the cells were washed twice with DMEM with gentle rocking. The supernatant was removed, with the pellet containing the cells resuspended in DMEM containing 10% FBS. At 24- and 48 h post-infection, the supernatant was collected, mixed, and filtered through the 0.45-µm filter unit (Millipore, MA, USA). Vero cells (1 × 10^5^ cells/well) grown in a 96-well plate were challenged with 15 µL of the pseudotype luciferase reporter virus and incubated at 37°C for 15 h. Luciferase activity was measured using a Luciferase Assay System (Promega, WI, USA).

### Fusion assay

VeroE6-TMPRSS2 cells were seated in a 24-well plate at a cell density of 2 × 10^5^ cells/well (Corning). The cells were transfected with 0.5 µg of the plasmid coding the S protein codon optimized to express in the mammalian cell lines under the CAG promotor and 0.5 µg of the plasmid coding EGFP under the CMV promotor by TransIT-LT1 transfection reagent (Mirus). At 10 h post-transfection, the cells were fixed in phosphate-buffered saline (PBS) containing 4% paraformaldehyde (Nacalai Tesque). Nuclei were stained with Hoechst (Thermo Fisher Scientific). The syncytia were counted.

### qRT-PCR

RNA was extracted from each cell line using Sepasol-RNA I Super G (Nacalai Tesque). The extracted RNA was reverse transcribed into cDNA using ReverTra Ace (Toyobo, Osaka, Japan) and random hexamers (Thermo Fisher Scientific). cDNA was quantified by qPCR using the Fast SYBR Green Master Mix (Thermo Fisher Scientific). Primer sets used are listed in S1 Table.

### Inhibitory assay

Vero cells cultured in a 96-well plate at a cell density of 1 × 10^5^ cells/well were treated with 1% dimethyl sulfoxide (Sigma-Aldrich) or 100 µM decanoyl-RVKR-CMK (Cayman Chemical, MN, USA) and 50 µM E64d (Calbiochem, Darmstadt, Germany) or 50 µM nafamostat (Cayman Chemical), or both, for 1 h. The cells were challenged with 15 µL of pseudotype virus or 50 PFU of recombinant virus and incubated for 15 and 20 h, respectively. Luciferase activity was measured using the Luciferase Assay System (Promega) for pseudotype viruses. The infected cells were visualized by an indirect fluorescence assay using an anti-SARS-CoV-2 N protein antibody (a kind gift by Dr. Okamoto) and anti-mouse IgG antibody-CF488 conjugate (Nacalai Tesque).

### Circular polymerase extension reaction

The reverse genetics system for rescuing recombinant SARS-CoV-2 using CPER has been described previously (36). As the first propagated recombinant viruses isolated from the HEK293-3P6C33 cells did not show a sufficient viral titer, the rescued virus was inoculated onto VeroE6-TMPRSS2 cells, and the supernatant was collected at 2 dpi and used as a viral stock.

### Virus titration

Vero cells were seated on a 12-well plate at a cell density of 2 × 10^5^ cells/well. The SARS-CoV-2 solution was diluted in DMEM containing 2% FBS. Approximately 100 µL of this diluted virus solution was inoculated onto Vero cells and incubated at 37°C for 1 h. After incubation, the cells were washed with PBS twice and layered with DMEM containing 2.5% FBS and 0.6% methylcellulose (FUJIFILM Wako Pure Chemical, Osaka, Japan). The plate was incubated at 37°C until plaques were observed in the well, and the cells were fixed in PBS containing 10% formaldehyde (FUJIFILM Wako Pure Chemical). The cells were stained with crystal violet (FUJIFILM Wako Pure Chemical), and plaques were counted.

### Western blotting

Vero cells were seated on a 12-well plate at a cell density of 2 × 10^5^ cells/well. Cells were infected with SARS-CoV-2 at an MOI of 1 and incubated at 37°C for 24 h. The cultured cells were subjected to centrifugation at 1,600 × *g* for 5 min. The supernatant was removed, and the cells were lysed using 1 × SDS sample buffer. The lysed cells were boiled at 95°C for 3 min, sonicated using Bioruptor II (BM Equipment, Tokyo, Japan), and centrifuged at 16,400 × *g* for 3 min. The samples were loaded onto a 10% TGX FastCast Acrylamide gel (Bio-Rad, CA, USA) and electrophoresed. Then, the proteins were transferred to a polyvinylidene difluoride membrane (Millipore). The membrane was blocked with PBS containing 0.05% tween-20 (PBS-T; Nacalai Tesque) and 5% skimmed milk (Nacalai Tesque) and reacted with rabbit anti-SARS-CoV-2 S1 (clone: SA39; Cell Engineering Corporation, Osaka, Japan) or mouse anti-S2 monoclonal antibodies (GeneTex, CA, USA). The membrane was washed with PBS-T twice and reacted with HRP-conjugated anti-mouse or rabbit IgG antibodies (Sigma-Aldrich). Protein bands were visualized using Chemi-Lumi One Ultra (Nacalai Tesque) and Amersham ImageQuant 800 (GE Healthcare, IL, USA).

### Inactivation and purification of SARS-CoV-2

Approximately 200 mL of SARS-CoV-2 solution was reacted with 0.025% b-propiolactone (FUJIFILM Wako Pure Chemical) at 4°C for 24 h. After 24 h, the reaction mix was incubated at 37°C for 2 h. The reaction mix was loaded onto PBS containing 20% sucrose (Nacalai Tesque) as a cushion and ultracentrifuged at 174,900 × *g* at 4°C for 2 h. The pellet was resuspended in PBS. The suspension was gently loaded onto a sucrose gradient (15%–60%) and ultracentrifuged at 153,700 × *g* at 4°C for 2 h. The viral bands were visualized in an ultracentrifuge tube and collected. The purified virus was mixed with 3.5–4.5 mL of PBS and ultracentrifuged at 174,900 × *g* at 4°C for 2 h. The pellet was resuspended in PBS, and the protein concentration was measured using Pierce BCA Protein Assay Kit (Thermo Fisher Scientific).

### Animal experiments

The Syrian hamsters (Mesocricetus auratus) were purchased from Japan SLC, Shizuoka, Japan. All animal experiments were conducted using 4-week-old male hamsters. The immunization of inactivated SARS-CoV-2 was performed by intramuscular inoculation of 6 µg of inactivated antigen per 2 weeks. Immunization was conducted twice. Immunized and unimmunized hamsters were administered intranasally with 1 × 10^5^ PFU of all SARS-CoV-2 strains, except for the Delta strain (1 × 10^4^ PFU). The body weights of the hamsters were measured daily. At five dpi, the hamsters were euthanized, and the left lung was collected to measure lung weight. The right lung used for viral titration was homogenized using Multi Beads Shocker (Yasui Kikai, Osaka, Japan) and centrifuged at 1,600 × *g* at 4°C for 5 min. The lungs used for histopathological analysis were immediately soaked in PBS containing 10% formalin. During all experiments, euthanasia was not performed until 5 dpi, owing to the sound health of all animals.

### Histopathological analyses

The lungs of the hamsters were collected, fixed in 10% neutral buffered formalin, and processed routinely to prepare paraffin-embedded tissue sections. Approximately 4.0 µm sections were cut and stained routinely with hematoxylin and eosin (HE) for histopathologic examination. Image analysis of HE-stained tissue sections was performed using QuPath v0.4.3 software. Immunohistochemical staining for SARS-CoV-2 nucleocapsid protein was performed as follows. Tissue sections of 4 µm thickness were deparaffinized and heated at 121°C for 15 min in antigen retrieval buffer, pH 6.0 (Nichirei Bioscience). After washing with PBS, endogenous peroxidase was quenched with 3% hydrogen peroxide in PBS. After blocking with 5% skimmed milk in PBS for 30 min, the tissue sections were incubated at 4°C overnight with anti-SARS-CoV-2 nucleocapsid protein (clone: 3A9, 1:500; Cell Engineering Corporation). After washing with PBS, the sections were incubated with Histofine Simple Stain Mouse MAX PO (Nichirei Bioscience, Tokyo, Japan) for 30 min at 20–25°C. Positive signals were visualized using peroxidase-diaminobenzidine, and the sections were counterstained with hematoxylin.

### Statistical analyses

Two-way analysis of variance (ANOVA) was used to determine the significant differences in growth kinetics and loss of body weight in SARS-CoV-2-affected hamsters. Welch’s *t*-test and one-way ANOVA were used for all other experiments. Statistical analyses were conducted using the GraphPad Prism 9 software (GraphPad Software).

## Data Availability

The viral genome sequences of the SARS-CoV-2 Kng P30 and B-1 P30 strains (bulk) have been deposited in GISAID (https://www.gisaid.org/) under the accession numbers EPI_ISL_17642389 and EPI_ISL_17642388, respectively.

## SUPPLEMENTAL MATERIAL

Fig. S1 spectrum.02859-23-s0001.TIF Virological analysis of recombinant severe acute respiratory syndrome coronavirus 2 (SARS-CoV-2) strains bearing single or multiple mutations.

Fig. S2 spectrum.02859-23-s0002.TIF Western blotting of infected cells with ΔCS recombinant SARS-CoV-2 strain.

Fig. S3 spectrum.02859-23-s0003.TIF Cell-cell fusion activity of the Spike (S) protein of Vero cell-adapted SARS-CoV-2 strains.

Fig. S4 spectrum.02859-23-s0004.TIF Histopathological analysis of hamsters immunized with inactivated SARS-CoV-2 strains.

Table S1 spectrum.02859-23-s0005.TIF List of primer pairs used for qRT-PCR.

ASM does not own the copyrights to Supplemental Material that may be linked to, or accessed through, an article. The authors have granted ASM a non-exclusive, world-wide license to publish the Supplemental Material files. Please contact the corresponding author directly for reuse.
