# Supplementary figures and images for: Vero cell-adapted SARS-CoV-2 strain shows increased viral growth through furin-mediated efficient spike cleavage
Source: Microbiol Spectr. 2024 Feb 28;12(4):e02859-23. doi: 10.1128/spectrum.02859-23 (PMC10986611; doi:10.1128/spectrum.02859-23)

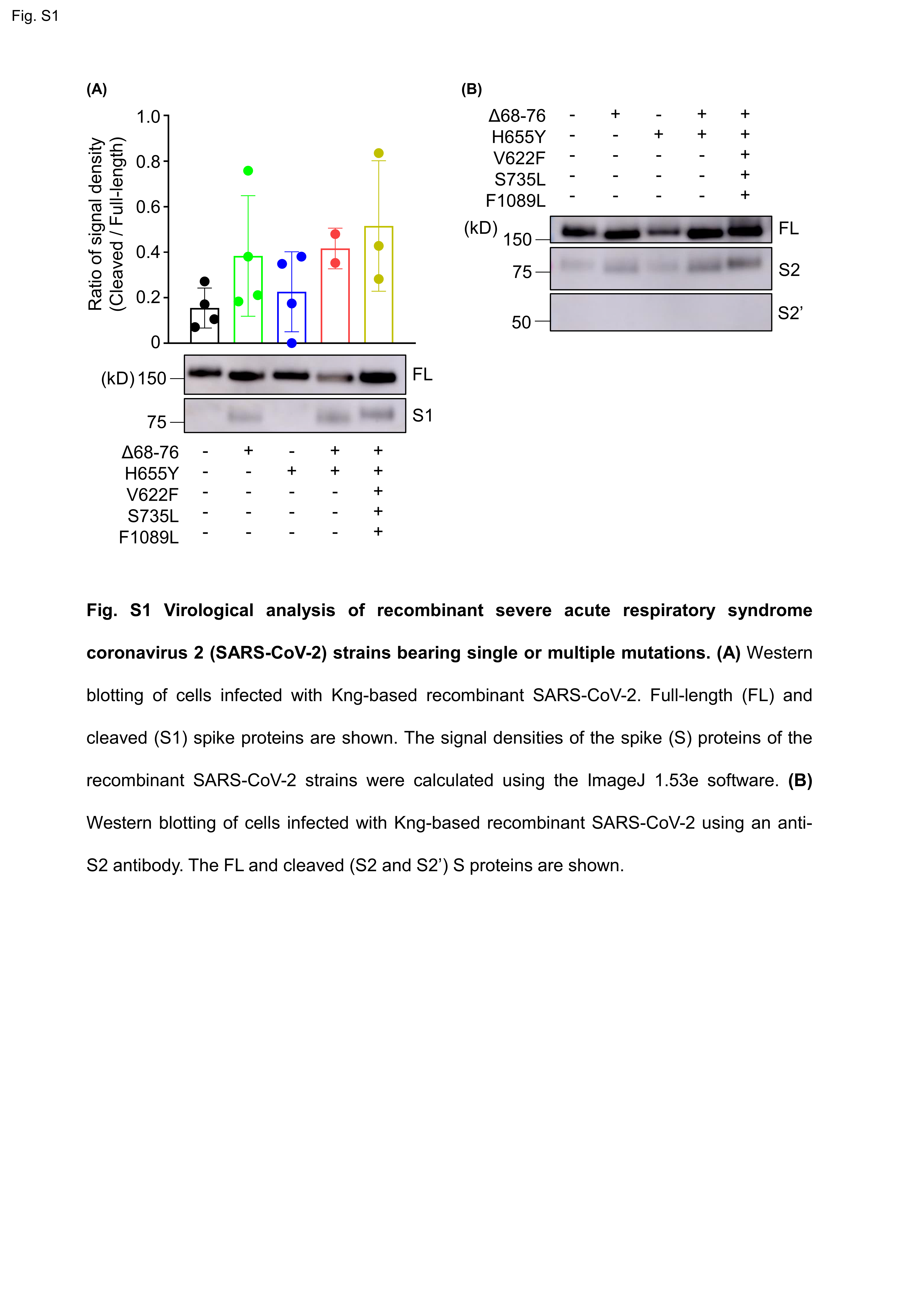

Supplement: Fig. S1 — Virological analysis of recombinant severe acute respiratory syndrome coronavirus 2 (SARS-CoV-2) strains bearing single or multiple mutations. [file spectrum.02859-23-s0001.tif]

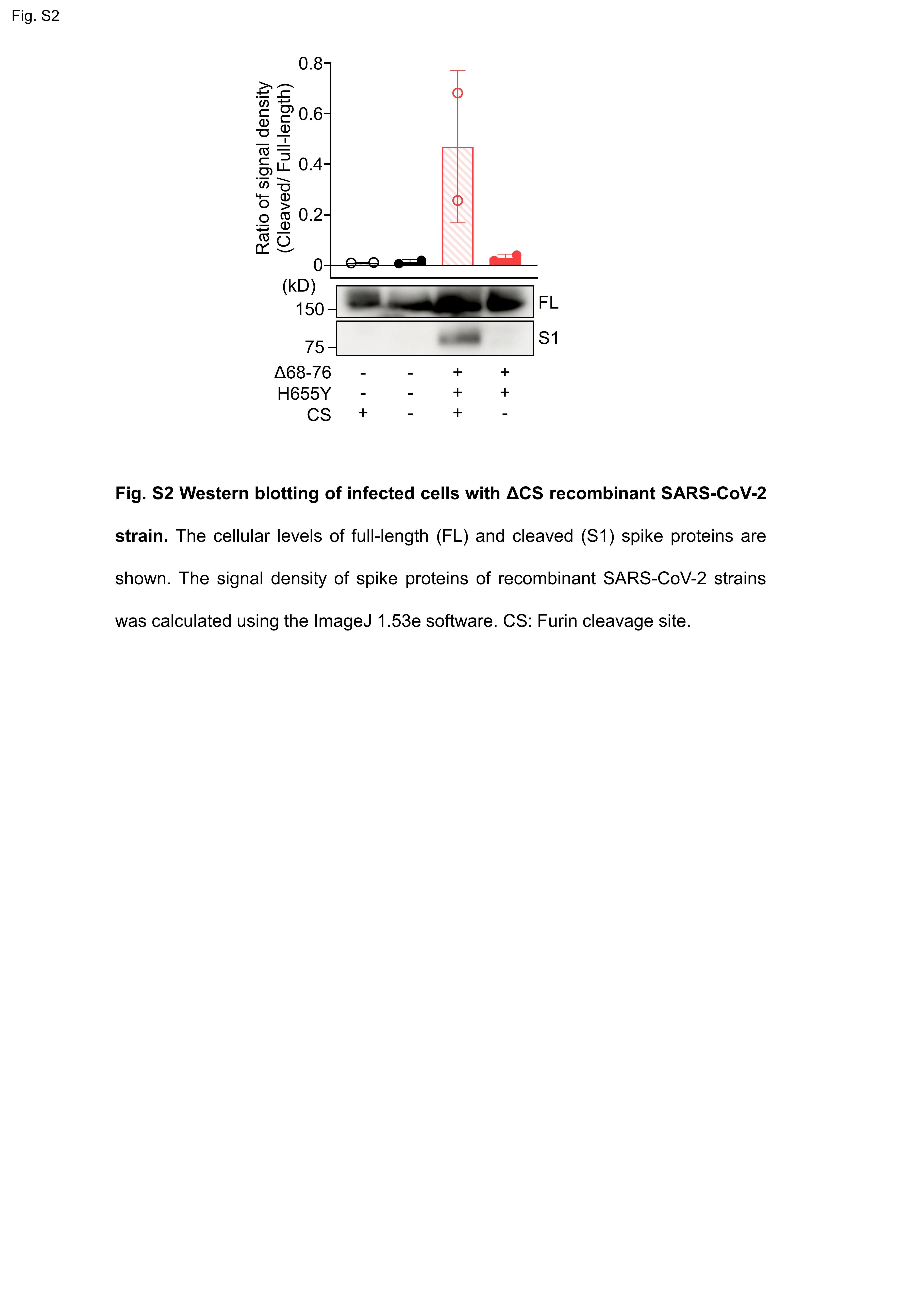

Supplement: Fig. S2 — Western blotting of infected cells with ΔCS recombinant SARS-CoV-2 strain. [file spectrum.02859-23-s0002.tif]

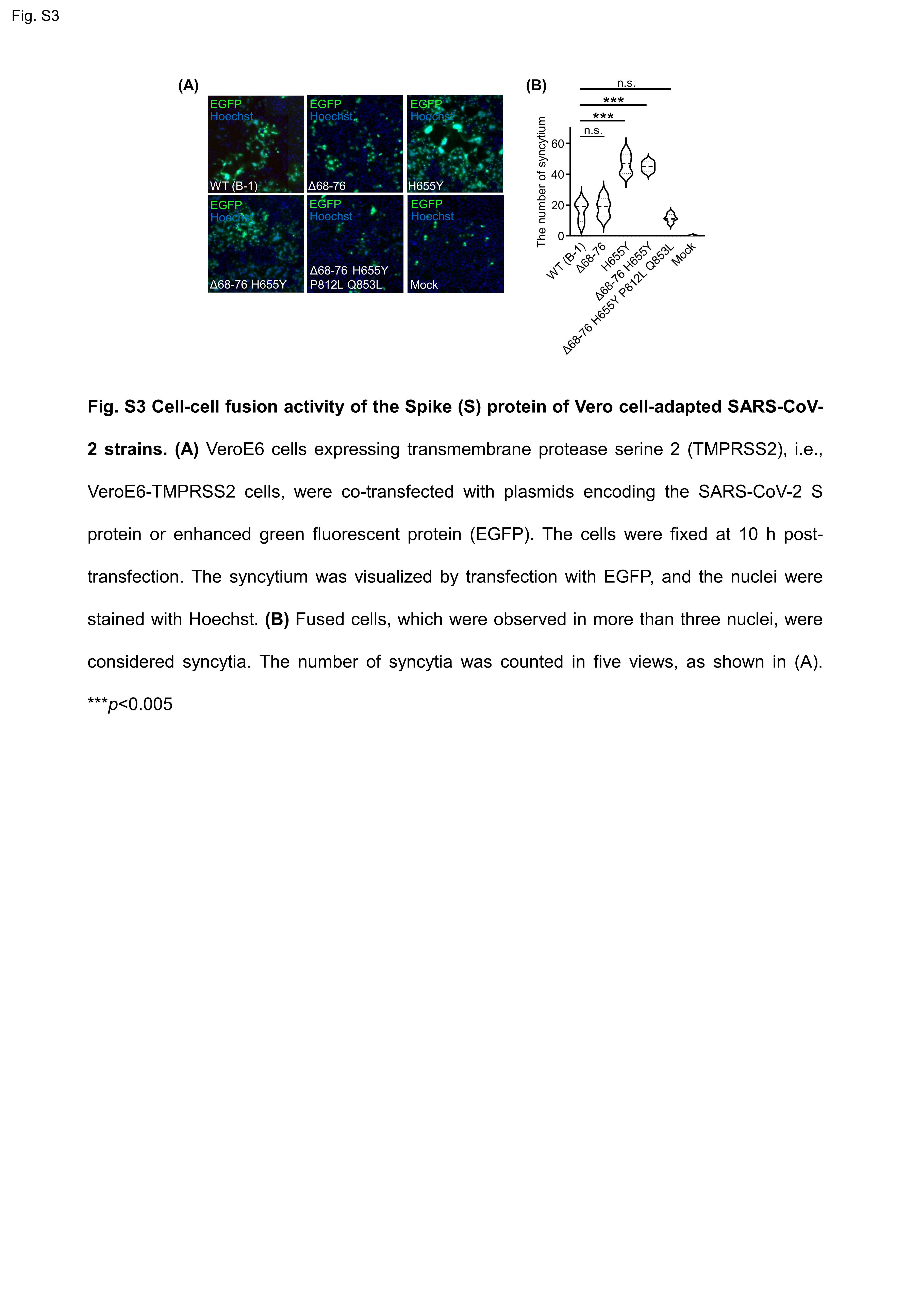

Supplement: Fig. S3 — Cell-cell fusion activity of the Spike (S) protein of Vero cell-adapted SARS-CoV-2 strains. [file spectrum.02859-23-s0003.tif]

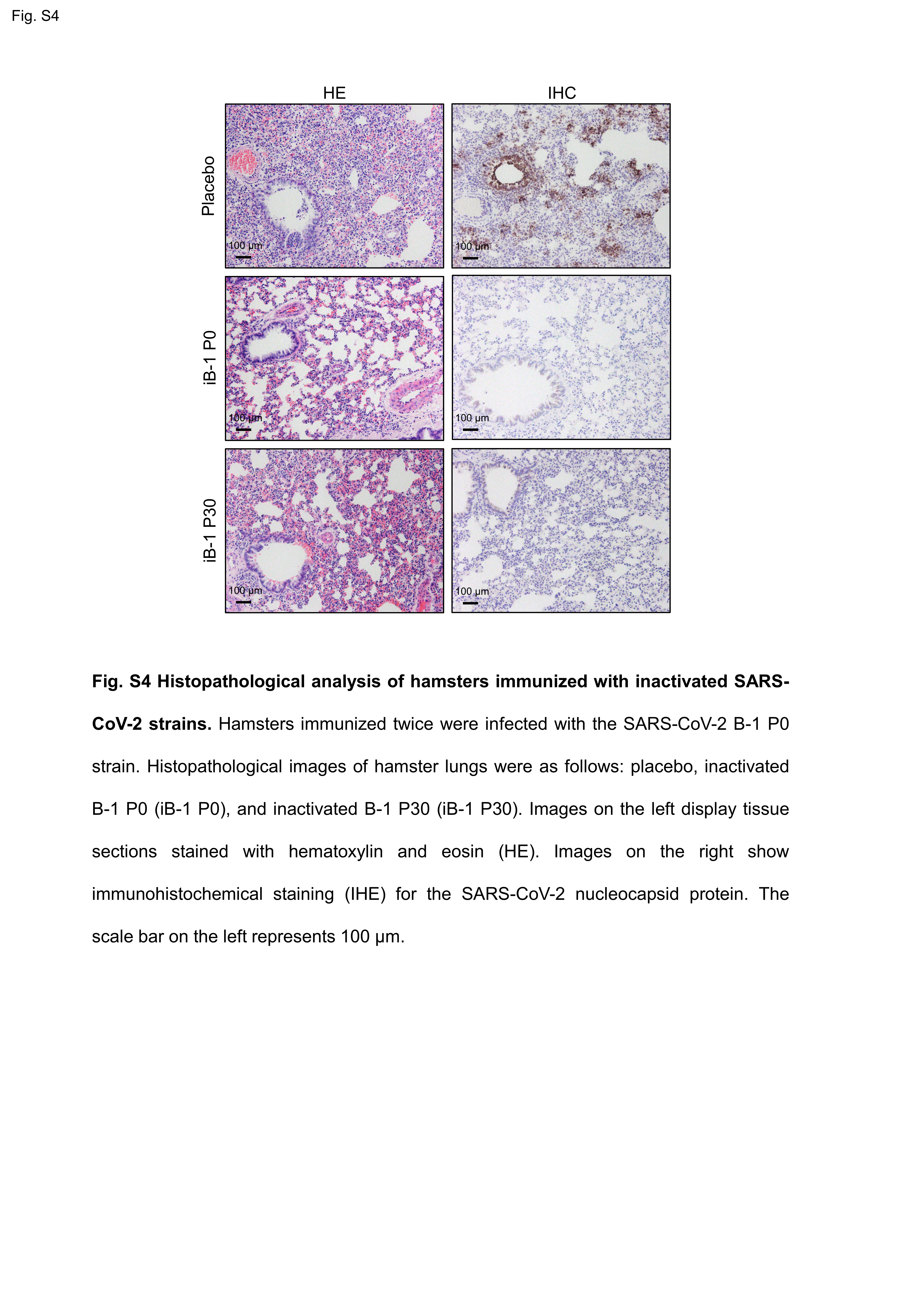

Supplement: Fig. S4 — Histopathological analysis of hamsters immunized with inactivated SARS-CoV-2 strains. [file spectrum.02859-23-s0004.tif]

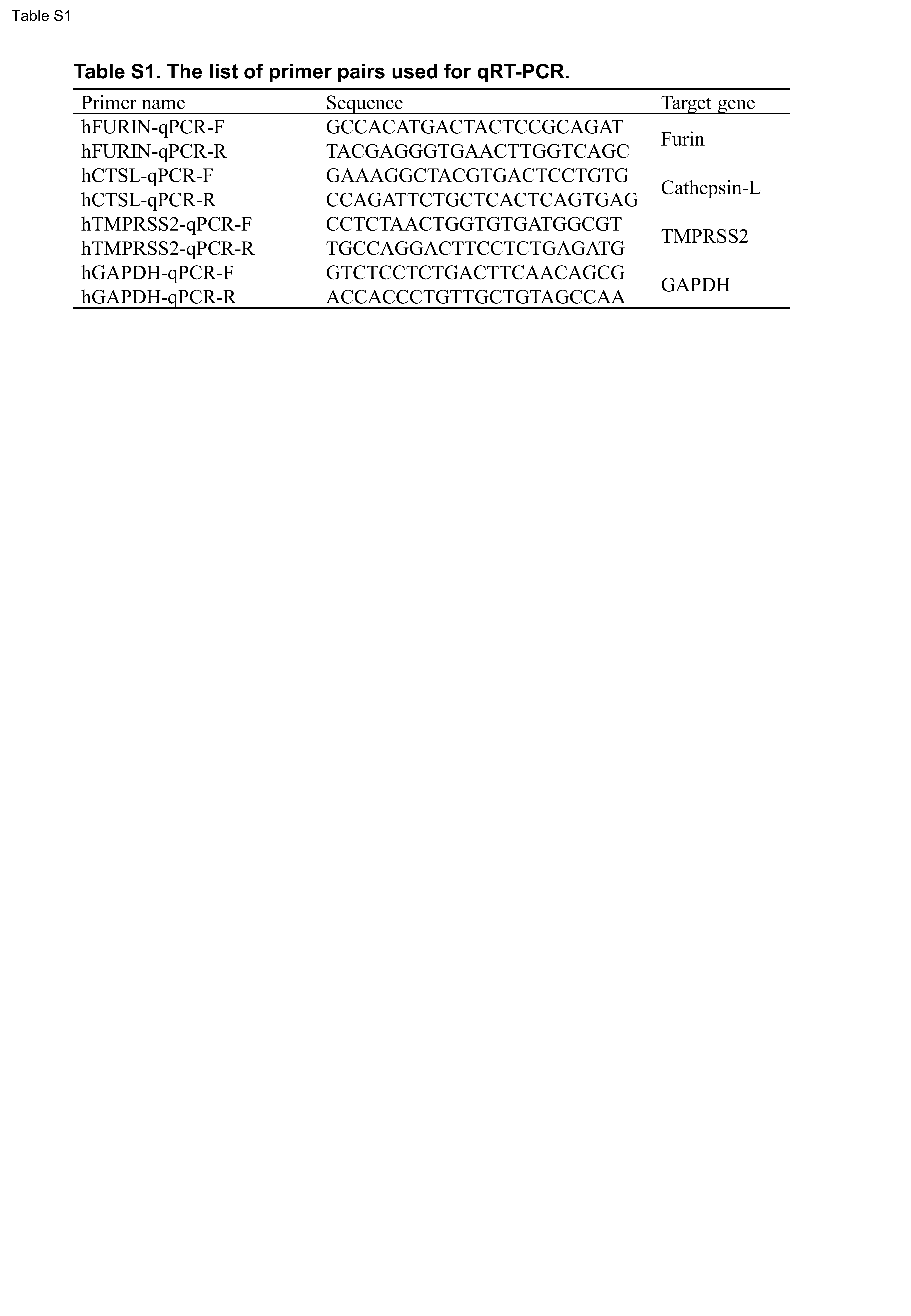

Supplement: Table S1 — List of primer pairs used for qRT-PCR. [file spectrum.02859-23-s0005.tif]
